# Unmasking Type 1 Brugada Pattern Following Pilsicainide Administration for Paroxysmal Atrial Fibrillation: A Case Report

**DOI:** 10.31662/jmaj.2025-0287

**Published:** 2025-09-19

**Authors:** Yasushi Matsushita, Naoyuki Otani, Takashi Tomoe, Satoshi Mizuguchi, Shoya Ono, Keijiro Kitahara, Takushi Sugiyama, Takanori Yasu, Yasuhiro Maejima

**Affiliations:** 1 Midorijuji Cinic Department of Cardiovascular Medicine, Yokohama, Japan; 2 Department of Cardiac and Vascular Surgery, Dokkyo Medical University Nikko Medical Center, Nikko, Japan; 3 Department of Cardiology, Dokkyo Medical University Nikko Medical Center, Nikko, Japan; 4 Department of Cardiology, National Hospital Organization, Yonago Medical Center, Yonago, Japan; 5 Department of Cardiovascular Medicine and Nephrology, Dokkyo Medical University Nikko Medical Center, Nikko, Japan

**Keywords:** Brugada syndrome, pilsicainide, drug-induced ECG, atrial fibrillation

## Abstract

Brugada syndrome is a genetic arrhythmia characterized by coved ST-segment elevation in the right precordial leads, predisposing individuals to sudden cardiac death due to fatal ventricular arrhythmias. Currently, a type 1 Brugada electrocardiographic (ECG) pattern can be used to diagnose the condition; however, diagnosis is difficult because normal intercostal spaces do not always allow for the detection of a type 1 Brugada ECG. Sodium channel blockers, including pilsicainide, a class Ic antiarrhythmic agent, cause Brugada-type ECG patterns. We report the case of a 79-year-old man with no prior syncope or family history of Brugada syndrome who developed a drug-induced type 1 Brugada ECG pattern after treatment for paroxysmal atrial fibrillation with pilsicainide. The patient presented with palpitations and atrial fibrillation, for which pilsicainide and rivaroxaban were administered. ECG performed after symptom resolution revealed a coved-type ST-segment elevation characteristic of type 1 Brugada ECG. The patient remained asymptomatic, and pilsicainide was immediately discontinued. Subsequent ECGs normalized within 3 days. No ventricular arrhythmias were observed during >6 months of follow-up, and an implantable cardiac monitor was placed for ongoing surveillance.

This case highlights the importance of recognizing drug-induced Brugada-type ECG patterns, particularly in asymptomatic patients treated with sodium channel blockers. Although the presence of a type 1 ECG pattern is diagnostic of Brugada syndrome, its prognostic significance in drug-induced asymptomatic cases remains uncertain. Existing data suggest that asymptomatic patients with drug-induced type 1 ECGs and no family history of or prior arrhythmic events generally have a favorable prognosis and do not require implantable cardioverter-defibrillator placement. This case highlights that even patients with normal ECGs may present with drug-induced Brugada-type patterns. Clinicians should always be alert to ECG changes when prescribing sodium channel blockers for atrial fibrillation.

## Introduction

Brugada syndrome is an inherited cardiac arrhythmia disorder characterized by a right bundle branch block (RBBB) pattern and persistent ST-segment elevation in the right precordial leads, often predisposing affected individuals to sudden cardiac death due to polymorphic ventricular tachycardia or fibrillation in the absence of structural heart disease ^(1), (2)^. A type 1 Brugada electrocardiographic (ECG) pattern, marked by a coved-type ST-segment elevation of ≥2 mm followed by a negative T wave, is diagnostic when spontaneously observed or induced by sodium channel blockers. In contrast, a saddleback-type ST-segment elevation of ≥1 mm is classified as type 2, while an elevation of <1 mm is classified as type 3. Pilsicainide unmasks latent Brugada patterns and is employed in diagnosing Brugada syndrome ^(3)^. We report a case wherein pilsicainide administration for paroxysmal atrial fibrillation accidentally unmasked a type 1 Brugada ECG pattern.

## Case Report

**Figure 1.**
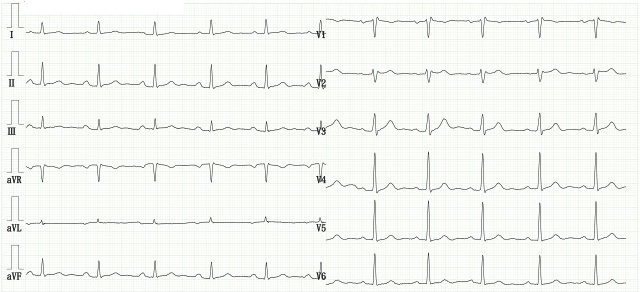
Electrocardiogram obtained 2 years before the current visit. The electrocardiogram shows a normal sinus rhythm with an incomplete right bundle branch block, a frequently observed finding. However, ST elevation in lead V2 is <1 mm, displaying a saddleback pattern characteristic of type 3 Brugada electrocardiographic pattern.

**Figure 2.**
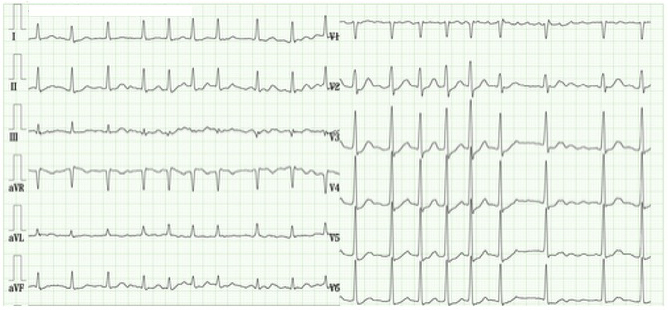
Electrocardiogram recorded during a visit with a complaint of palpitations. The electrocardiogram shows the presence of atrial fibrillation.

**Figure 3.**
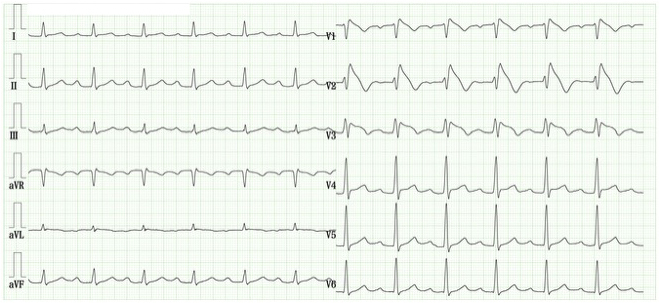
Electrocardiogram following pilsicainide administration for paroxysmal atrial fibrillation. The electrocardiogram demonstrates the restoration of normal sinus rhythm. However, lead V2 reveals a prominent coved-type ST-segment elevation measuring ≥2 mm, followed by an inverted T wave, consistent with a type 1 Brugada electrocardiographic pattern.

A 79-year-old male presented to our hospital with palpitations that persisted for several days. His medical history included vasospastic angina, dyslipidemia, and chronic constipation. He had been treated with diltiazem 100 mg, pitavastatin 1 mg, and magnesium oxide 1.2 g. A 12-lead ECG performed 2 years prior demonstrated a type 3 Brugada ECG pattern (Figure 1). At presentation, his vital signs were stable: blood pressure, 118/71 mmHg; heart rate, 110 bpm; and SpO_2_, 97% on room air. Initial ECG revealed atrial fibrillation with a ventricular response of 108 bpm (Figure 2). Laboratory tests were all within normal limits. Transthoracic echocardiography revealed a structurally normal heart. For pharmacological cardioversion, 150 mg oral pilsicainide and 15 mg rivaroxaban were administered. The following day, atrial fibrillation resolved, and a repeat ECG showed sinus rhythm along with a type 1 Brugada-type ECG pattern, presenting a typical coved-type ST-segment elevation in the right precordial leads (Figure 3). The patient remained asymptomatic with no episodes of syncope, seizures, or documented ventricular arrhythmias.

**Figure 4.**
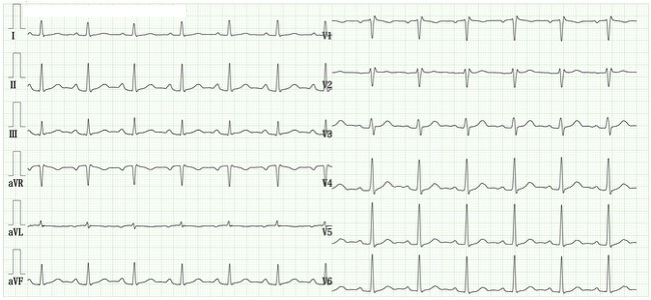
Electrocardiogram 4 days after pilsicainide administration. Restoration of sinus rhythm on the electrocardiogram following pilsicainide administration.

Pilsicainide was immediately discontinued. A follow-up ECG performed 4 days after drug cessation showed resolution of the type 1 pattern, returning to the baseline RSR’ pattern (Figure 4). Additional ECGs recorded at the higher intercostal spaces did not demonstrate type 1 Brugada features. The patient subsequently underwent catheter ablation for atrial fibrillation at another institution and was implanted with an implantable loop recorder for continuous arrhythmia monitoring. During follow-up (>6 months), no ventricular arrhythmia or syncope episodes were documented. Therefore, prophylactic implantable cardioverter-defibrillator placement was not performed, and conservative monitoring was used instead. The patient had no family history of sudden cardiac death or Brugada syndrome.

## Discussion

This case highlights the unmasking of a type 1 Brugada ECG pattern in an asymptomatic patient following pilsicainide administration for atrial fibrillation. The most serious clinical symptom in Brugada syndrome is sudden cardiac death due to ventricular fibrillation. While the average age of patients experiencing ventricular fibrillation is 41 ± 15 years, this patient was 79 years old and had no such episodes ^(4)^. At baseline, the patient showed a nonspecific type 3 Brugada ECG pattern. However, pilsicainide administration inadvertently revealed a type 1 Brugada ECG pattern, effectively mimicking a drug challenge test. Symptomatic patients―those with ventricular tachycardia, ventricular fibrillation, or syncope― who also exhibit a spontaneous type 1 Brugada pattern are at high risk for sudden cardiac death. In contrast, asymptomatic patients with no family history and a drug-induced type 1 pattern are generally at low risk ^(2)^. In a Japanese cohort, none of the 46 asymptomatic individuals with drug-induced type 1 ECGs experienced life-threatening arrhythmias ^(5)^. Similarly, the FINGER Brugada syndrome registry found that among 386 asymptomatic patients with drug-induced type 1 ECGs, only four experienced cardiac events, in contrast to 15 events in 175 symptomatic patients ^(6)^. Incomplete RBBB, observed in approximately 3% of the general population, can present with ECG features resembling type 2 or type 3 Brugada patterns, complicating Brugada syndrome diagnosis ^(7)^.

Atrial fibrillation is commonly observed in patients with Brugada syndrome ^(4)^, with a reported prevalence of 11% to 14%. Class Ic antiarrhythmic agents have been shown to enable a type 1 Brugada ECG pattern. In a study by Occhetta et al. ^(8)^, 34% of patients with a type 2 or 3 Brugada ECG pattern developed a type 1 pattern following pharmacological intervention. Given the prevalence of atrial fibrillation in older patients and their known association with Brugada syndrome ^(9)^, clinicians should maintain vigilance when prescribing sodium channel blockers. This case reinforces the necessity for ECG monitoring before and after initiating such agents. It also emphasizes that drug-induced type 1 Brugada ECG without symptoms or family history warrants observation rather than aggressive treatment.

## Article Information

### Acknowledgments

We would like to thank Editage (www.editage.jp) for English language editing.

### Author Contributions

Concept: Yasushi Matsushita and Naoyuki Otani. Data collection: Yasushi Matsushita, Naoyuki Otani. Discussion: Yasushi Matsushita, Naoyuki Otani, Takashi Tomoe, Satoshi Mizuguchi, Shoya Ono, Keijoro Kitahara, Takushi Sugiyama, Takanori Yasu, and Yasuhiro Maejima. All authors have read and agreed to the published version of the manuscript.

### Conflicts of Interest

None

### Informed Consent

Written informed consent for publication was obtained from the patient.
